# Eicosapentaenoic acid and docosahexaenoic acid reduce interleukin-1β-mediated cartilage degradation

**DOI:** 10.1186/ar3183

**Published:** 2010-11-08

**Authors:** Angus KT Wann, Jiten Mistry, Emma J Blain, Adina T Michael-Titus, Martin M Knight

**Affiliations:** 1 School of Engineering and Materials Science, Queen Mary University of London, Mile End, London, E1 4NS, UK; 2 Blizzard Institute of Cell and Molecular Sciences, Queen Mary University of London, 4 Newark Street, London, E1 2AT, UK; 3 Arthritis Research UK Biomechanics and Bioengineering Centre, Cardiff School of Biosciences, Cardiff University, Museum Avenue, Cardiff, CF10 3AX, UK

## Abstract

**Introduction:**

In inflammatory joint disease, such as osteoarthritis (OA), there is an increased level of proinflammatory cytokines, such as interleukin (IL)-1β. These cytokines stimulate the production of matrix metalloproteinases (MMPs), which leads to the degradation of the cartilage extracellular matrix and the loss of key structural components such as sulphated glycosaminoglycan (sGAG) and collagen II. The aim of this study was to examine the therapeutic potential of n-3 polyunsaturated fatty acids (PUFAs) in an *in vitro *model of cartilage inflammation.

**Methods:**

Two specific n-3 compounds were tested, namely, eicosapentaenoic acid (EPA) and docosahexaenoic acid (DHA), each at 0.1, 1 and 10 μM. Full thickness bovine cartilage explants, 5 mm in diameter, were cultured for 5 days with or without IL-1β and in the presence or absence of each n-3 compound. The media were replaced every 24 hours and assayed for sGAG content using the 1,9-dimethylmethylene blue (DMB) method. Chondrocyte viability was determined at the end of the culture period using fluorescence microscopy to visualise cells labelled with calcein AM and ethidium homodimer.

**Results:**

Treatment with IL-1β (10 ng.ml^-1^) produced a large increase in sGAG release compared to untreated controls, but with no effect on cell viability, which was maintained above 80% for all treatments. In the absence of IL-1β, both n-3 compounds induced a mild catabolic response with increased loss of sGAG, particularly at 10 μM. By contrast, in the presence of IL-1β, both EPA and DHA at 0.1 and 1 μM significantly reduced IL-1β-mediated sGAG loss. The efficacy of the EPA treatment was maintained at approximately 75% throughout the 5-day period. However, at the same concentrations, the efficacy of DHA, although initially greater, reduced to approximately half that of EPA after 5 days. For both EPA and DHA, the highest dose of 10 μM was less effective.

**Conclusions:**

The results support the hypothesis that n-3 compounds are anti-inflammatory through competitive inhibition of the arachidonic acid oxidation pathway. The efficacy of these compounds is likely to be even greater at more physiological levels of IL-1β. Thus we suggest that n-3 PUFAs, particularly EPA, have exciting therapeutic potential for preventing cartilage degradation associated with chronic inflammatory joint disease.

## Introduction

Articular cartilage allows relatively frictionless motion of the diarthrodial joint and serves as a unique load-bearing material. The properties of the tissue are related to the composition and structure of the extracellular matrix, which is maintained by the chondrocytes. This hydrated matrix consists of proteoglycan aggregates, containing sulphated glycosaminoglycans (sGAG), which are entangled in a meshwork of collagen, predominantly type II. Chondrocytes synthesise the various matrix components as well as enzymes which lead to their catabolism (aggrecanases and matrix metalloproteinases, or MMPs) and inhibitors of these enzymes, namely, tissue inhibitors of metalloproteinases (TIMPs). In healthy tissues, a balance is maintained between matrix synthesis and degradation.

Osteoarthritis (OA) is characterised by the progressive breakdown of the cartilage extracellular matrix. Increased aggrecan cleavage by aggrecanases leads to the loss of entrapped sulphated GAG and surrounding type II collagen, leading ultimately to joint cartilage destruction and the exposure of underlying bone [1-4].

Inflammatory cytokines, including interleukin (IL)-1β, have been detected in the synovial fluid of OA patients in the 1-4 pg/ml range [5]. It is well established that IL-1β stimulates cartilage breakdown [6,7]. Consequently numerous *in vitro *studies have used this cytokine in models of inflammatory OA [8-12]. Such studies have highlighted the procatabolic effects of IL-1 by measuring the expression of inflammatory markers such as MMP levels and cyclooxygenase (COX) and the release of matrix degradative products [13,14]. An understanding of inflammatory progression in the tissue, especially matrix degradation, exists, but the development of successful therapeutic interventions to halt this destructive process is in its infancy.

Certain polyunsaturated fatty acids (PUFAs) including eicosapentaenoic acid (EPA) and arachidonic acid (AA), n-3 and n-6 fatty acids, respectively, are "essential" to the health of mammals. Indeed, dietary PUFAs of both the n-3 and n-6 series have long been heavily implicated in human health [15]. Long chain n-3 fatty acids are present in fish and marine mammals, and epidemiological data indicate a correlation between fish-wealthy diets and reduced incidence of inflammatory disease [16]. It has been postulated that humankind evolved on a diet with a ratio of n-6:n-3 fatty acids of approximately 1:1, whereas, at least in Western society, the prevailing ratio in modern times is 10-20:1 [17]. In a variety of conditions, including neurological damage [18], cardiovascular disease [19] and rheumatoid arthritis [20] n-3 compounds have been shown to offer therapeutic benefit. Recent animal studies have also reported that n-3 fatty acids may have therapeutic benefit in OA [21]. This may be associated with increased collagen synthesis and decreased amounts of the inflammatory mediator prostaglandin E2 as reported in fibroblasts *in vitro *[22].

In general, the two series of fatty acids are thought to have opposing effects in the context of inflammation. Oxidation of n-6 fatty acids by the COX enzyme system gives rise to proinflammatory "2" series eicosanoids, such as the prostaglandin PGE_2_, and "4" series leukotrienes such as LTB_4_. By contrast, oxidation of n-3 series members give rise to the lesser inflammatory molecules of PGE_3_, following COX oxidation, and LTB_5_, following lipooxygenase activity [23]. The resultant competitive inhibition of AA oxidation represents the primary theory by which n-3 fatty acids may elicit therapeutic anti-inflammatory effects [23].

Here we test the hypothesis that the long-chain n-3 fatty acids, eicosapentaenoic acid (EPA) and docosahexaenoic acid (DHA), reduce cytokine-induced articular cartilage degradation as characterised by the release of sGAG from the tissue.

## Materials and methods

### Reagents and media preparation

Dulbecco's modified Eagle's medium (DMEM) was supplemented with 1.6 mM L-glutamine, 81 U/ml penicillin, 80 μg/ml streptomycin, 16 mM 2-[4-(2-hydroxyethyl)piperazin-1-yl]ethanesulfonic acid (or HEPES) buffer, and 0.68 mM ascorbic acid. This medium was used either on its own or with the addition of 16% (vol/vol) foetal calf serum (FCS). All of the above reagents were from Sigma-Aldrich (Poole, UK). IL-1β was obtained from Peprotech (London, UK) and was reconstituted to 1 mg.mL^-1 ^from solid lyophilised sterile powder in distilled water following centrifugation. This stock was then added to serum-free supplemented DMEM and frozen down in aliquots at 10 μg.mL^-1^. When required, aliquots of IL-1β were thawed and diluted to 10 ng.ml^-1 ^in serum-free medium containing bovine serum albumin (BSA; Sigma-Aldrich) at a final concentration of 0.1%. The fatty acids, *cis*-5,8,11,14,17-eicosapentaenoic acid (EPA) and *cis*-4,7,10,13,16,19-docosahexaenoic acid (DHA) were also obtained from Sigma-Aldrich and prepared into 1 M solutions in ethanol. These stock solutions were mixed 1:1 with 30% fatty acid free BSA (Sigma-Aldrich, Poole, UK) in phosphate-buffered saline (PBS) and diluted with serum-free medium to obtain aliquots at 100 μM which were frozen. Aliquots were freshly defrosted on the day of use and diluted in serum-free medium to concentrations of 0.1, 1 and 10 μM for both EPA and DHA. Fatty acid concentrations that were an order of magnitude above those used in this study are readily achievable in the plasma compartment following nutritional supplementation [24]. Here the stock solutions and resultant medium were not filtered after addition of the compounds to ensure dose accuracy. Vehicle concentrations tested were therefore 0.1% albumin and 0.0789, 0.789 and 7.89 μM ethanol.

1,9-dimethylmethylene blue (DMB) powder (Sigma-Aldrich) was reconstituted in ethanol and then in a 29 mM sodium formate/distilled water solution and finally the pH was adjusted to 3. DMB was used at a final concentration of 16 μg.mL^-1^. Chondroitin sulphate (6-sulphate:4-sulphate; 0.33:1) standards (Sigma-Aldrich) were made up at 0-50 μg.mL^-1 ^in the appropriate treatment medium for the sample being assayed.

### Tissue explant isolation and culture

**Figure 1:**
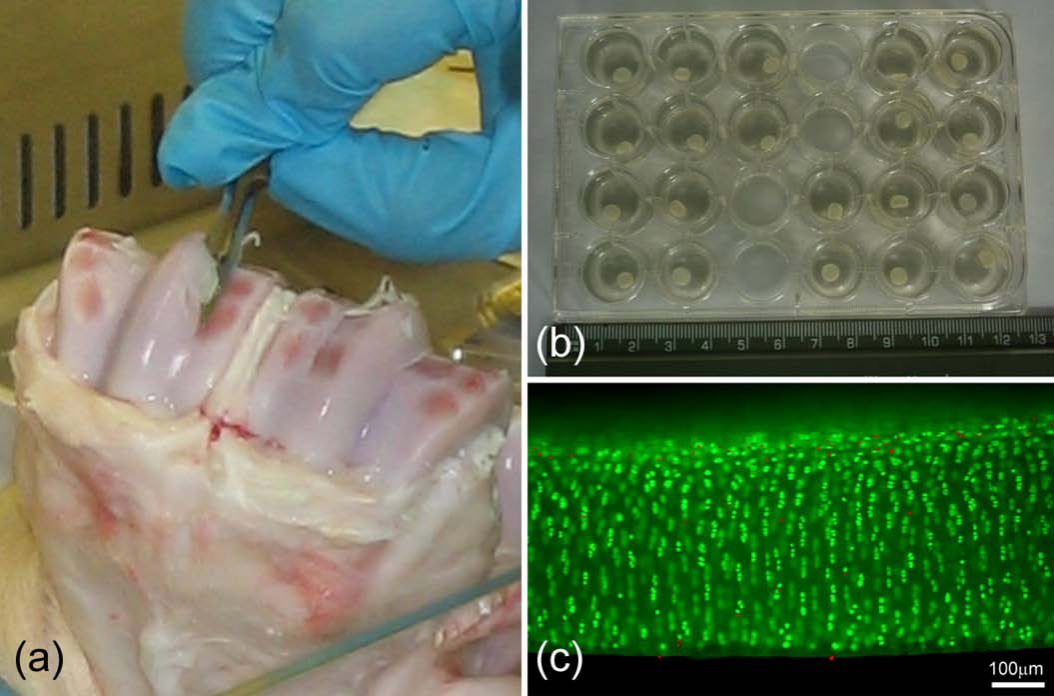
**Chondrocyte viability is maintained in cartilage explants taken from the bovine metacarpal phalangeal joint**. Full-depth cartilage explants were removed from the proximal surface of bovine metacarpal phalangeal joints **(a) **and cultured in 24-well plates with 2 ml of culture medium/explant/well **(b)**. Cell viability was assessed at the end of the culture period using fluorescence microscopy. Full-depth slices were removed from the explant and incubated in calcein AM, which labels live cells green, and ethidium homodimer, which labels dead cells red **(c)**.

Eight to ten bovine forefeet from 18-month-old steers were obtained fresh from slaughter from a local abattoir. Full-depth tissue explants were taken from the proximal surface of the metacarpalphalangeal joint under sterile conditions. The circular explants, 5 mm in diameter, were cut out by slowly rotating a dermal punch (Miltex, York, PA, USA) down through to the subchondral bone and removing them with a scalpel blade (Figure 1a). Explants were immediately placed into DMEM plus FCS and incubated at 37°C in 5% CO_2_. After 24 hours, explants were weighed and placed in 2 ml of fresh DMEM plus FCS in separate wells for 72 hours (Figure 1b). At 96 hours, the explants were thoroughly washed (3 × 5 min) to remove any serum. Groups of explants (n = 10) were then cultured for 120 hours in serum-free medium in the presence or absence of IL-1β (10 ng.mL^-1^) and supplemented with or without either EPA or DHA. The EPA and DHA were used at three different concentrations, namely, 0.1, 1 and 10 μM. An untreated control group was cultured in serum-free medium. Medium was removed every 24 hours for subsequent biochemical analysis and replaced with the appropriate fresh medium. Separate studies were conducted to examine the influence of the two vehicles for IL-1β and the fatty acids, namely, BSA and ethanol. The final concentrations of each vehicle are given in Table 1.

**Table 1:** Final concentrations of vehicles in the eight treatment groups^a^

	Bovine serum albumin (%)	Ethanol (μM)
IL-1 only	0.1	0
IL-1 + 0.1 μM n-3 compound	0.100003	0.0789
IL-1 + 1 μm n-3 compound	0.10003	0.789
IL-1 + 10 μm n-3 compound	0.1003	7.89
0.1 μM n-3 compound	0.000003	0.0789
1 μm n-3 compound	0.00003	0.789
10 μm n-3 compound	0.0003	7.89
Untreated control	0	0

^a^IL-1, interleukin 1.

For simplicity, any effect of the BSA used in the preparation of the n-3 compounds, EPA or DHA, was assumed to be negligible compared to the much greater concentration used for IL-1β (0.1%). Thus vehicle studies examined the effects of BSA at 0.1% and ethanol at 0.0789, 0.789 and 7.89 μM.

### Tissue viability

At the end of the culture period, one explant from each group was used to assess cell viability. The explant was incubated in a solution of 5 μM calcein AM and 5 μM ethidium homodimer (Invitrogen, Paisley, UK) for 60 minutes at 37°C. Full-depth sections 1 mm in thickness were cut using a scalpel, mounted on a fluorescence microscope and visualised with a × 20 objective. Live and dead cells are labelled green and red by calcein AM and ethidium homodimer, respectively. Percentage cell viability was calculated from at least 10 fields of view.

### Sulphated glycosaminoglycan (sGAG) quantification

The quantity of sGAG release into media was quantified in triplicate using the 1,9-dimethylmethylene blue (DMB) assay [25] with chondroitin sulphate calibration standards made up in media representing the treatment group being assayed. Samples were double- or triple-diluted, as necessary, to bring them into the range of the standards. Absorbance was read at 595 nm. The sGAG content was normalised to explant wet weight (approximately 20-30 mg).

As many data sets failed Kolmogorov-Smirnov normality testing, nonparametric statistical tests were used in the form of the Mann-Whitney *U *test and the Kruskal-Wallis test. Data are presented as median values with interquartile ranges for samples of 10 explants. All data and statistical analyses were conducted using GraphPad Prism 5 software (GraphPad Software, La Jolla, CA, USA).

## Results

### Cell viability

Mean cell viability for each treatment group is presented in Table 2, based on images of cells stained with calcein AM and ethidium homodimer at the end of the culture period. None of the treatment protocols had a statistically significant effect on cell viability (*P *> 0.05; one-way analysis of variance, or ANOVA). In all cases, viability was maintained above 80%. The majority of cell death was observed in all groups at the explant surfaces.

**Table 2:** Cell viability measured at the end of the entire culture period for different treatments^a^

Treatment		% Cell viability (means ± standard deviation)	
		
		Without IL-1β	With IL-1β
Control		87 ± 11	89 ± 9

EPA	0.1 μM	90 ± 8	83 ± 10
	1.0 μM	91 ± 7	87 ± 7
	10 μM	83 ± 10	89 ± 8

DHA	0.1 μM	85 ± 8	86 ± 5
	1.0 μM	85 ± 9	88 ± 5
	10 μM	83 ± 9	87 ± 10

0.1% BSA (vehicle)		86 ± 5	

7.89 μM ethanol (vehicle)		89 ± 8	

^a^Values represent sample means ± standard deviations for n = 1 explants with at least 10 fields of view for each explant. EPA, eicosapentaenoic acid; DHA, docosahexaenoic acid; IL-1β, interleukin 1β; BSA, bovine serum albumin.

### Effect of IL-1β on sGAG release

**Figure 2:**
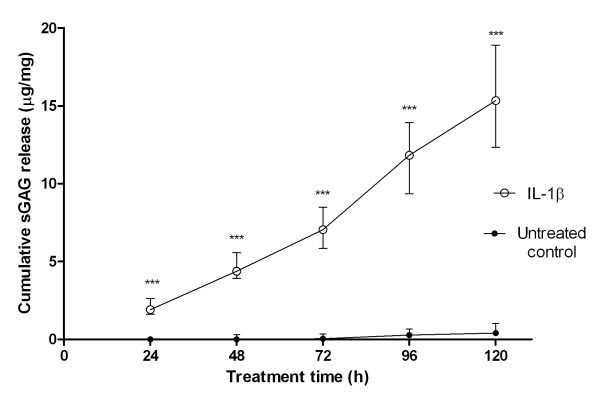
**Interleukin (IL)-1β produces cartilage degradation characterised by the release of sulphated glycosaminoglycan (sGAG)**. Temporal increase in cumulative sGAG release for untreated control explants and those cultured in the presence of 10 ng.ml^-1 ^interleukin. Values represent the median cumulative sGAG released from explants during the treatment period of 120 hours. Error bars indicate interquartile ranges. The sGAG release from IL-1-treated explants was statistically greater than that from untreated control explants at all time points (*P *< 0.001, n = 20; Mann-Whitney *U *test).

**Figure 3:**
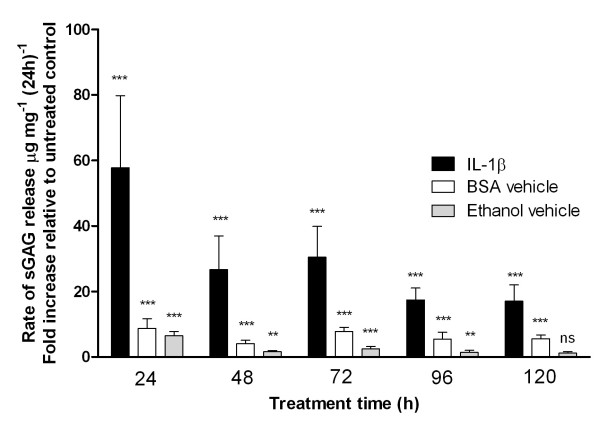
**The vehicles bovine serum albumin (BSA) and ethanol have a small catabolic effect**. Temporal changes in the release of sGAG for cartilage explants treated with either BSA or ethanol. Data are normalised to that for untreated control explants and presented as a fold increase with median values and error bars showing interquartile ranges. Data are grouped together for all ethanol doses (0.08-8 μM) as no statistically significant dose-response relationship was found. Statistically significant differences from untreated controls are indicated on the basis of Mann-Whitney *U *tests (*P < 0.05, **P < 0.01, ***P < 0.001).

Over the entire untreated culture period, sGAG was released from the cartilage explants into the culture media. Treatment with 10 ng.mL^-1 ^IL-1β produced a large increase in sGAG release, which was maintained over the 120-hour treatment period. Thus the cumulative sGAG released into the culture media was substantially greater than that for untreated controls at all time points (Figure 2), with all the differences being statistically significant (*P *< 0.001; Mann-Whitney *U *tests, n = 20). By the end of the culture period, after 120 hours of treatment, the IL-1β-treated explants showed a 38-fold increase in the amount of sGAG released compared with untreated controls. Although the absolute quantity of sGAG released per 24 hours increased over the 120-hour treatment period, the levels relative to untreated controls were greatest in the initial 24-hour period, decreasing thereafter (Figure 3).

### Influence of vehicles, BSA and ethanol

The vehicles used for IL-1β (0.1% BSA) and for EPA and DHA (ethanol) were also independently tested to evaluate their contributions. Figure 3 shows the amount of sGAG released into the medium per 24 hours over the 120-hour treatment period. Values have been normalised to untreated control values and are plotted alongside those for IL-1β-treated explants for comparison. Kruskal-Wallis tests revealed no consistent statistically significant differences between the three different ethanol concentrations (0.0789, 0.789 and 7.89 μM). The *P *values at time points 24, 48, 72, 96 and 120 hours were 0.42, 0.06, 0.02, 0.10 and 0.03, respectively. Hence these data for the separate ethanol concentrations have been pooled. Both vehicles, BSA and ethanol, produced increased sGAG release into the culture medium compared with untreated controls. The differences were statistically significant throughout the 120-hour treatment period, with the exception of ethanol over the 96- to 120-hour period.

### The effect of EPA and DHA on sGAG release rate from cartilage explants

**Figure 4:**
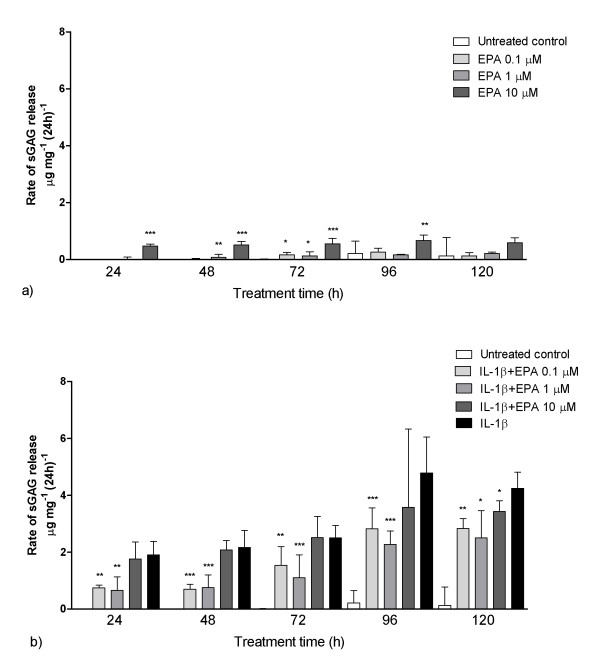
**Treatment with eicosapentaenoic acid (EPA) reduced the rate of cartilage degradation in the presence of IL-1β**. Over the 120 treatment period, sGAG release was measured in cartilage explants treated with EPA in the presence **(a) **and absence **(b) **of IL-1β. EPA was used at 0.1, 1 and 10 μM. Median data are shown with interquartile range error bars. Both sub-figures are on the same scale for comparative purposes. Statistically significant differences are shown relative to the untreated control group (a) and the IL-1β treatment group (b). Analysis was conducted using Mann-Whitney *U *tests (*P < 0.05, **P < 0.01, ***P < 0.001).

**Figure 5:**
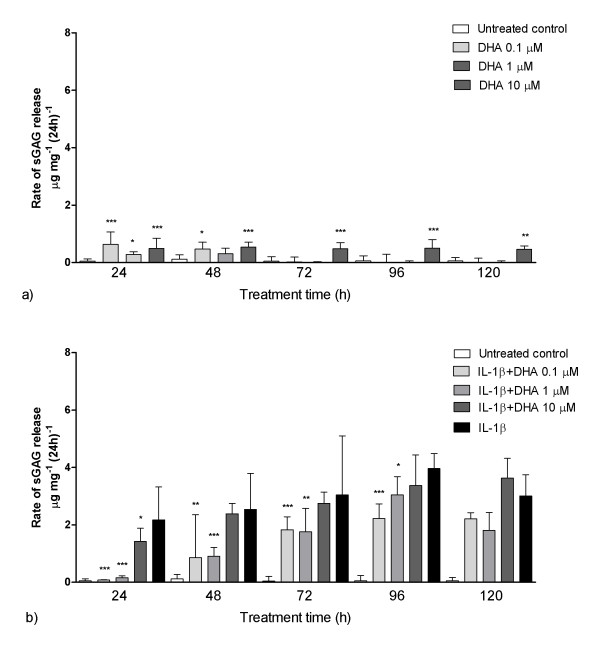
**Treatment with docosahexaenoic acid (DHA) reduced the rate of cartilage degradation in the presence of IL-1β**. Over the 120 treatment period, sGAG release was measured in cartilage explants treated with DHA in the presence **(a) **and absence **(b) **of IL-1β. DHA was used at 0.1, 1 and 10 μM. Median data are shown with interquartile range error bars. Statistically significant differences are shown relative to the untreated control group (a) and the IL-1β treatment group (b). Analysis was conducted using Mann-Whitney *U *tests (*P < 0.05, **P < 0.01, ***P < 0.001).

The effects of EPA and DHA on sGAG release rate in the presence and absence of IL-1β are shown in Figures 4 and 5. The data show the sGAG released into the culture media per 24 hours, normalised to the initial wet weight of the explant. In the absence of IL-1β, treatment with EPA produced a slight increase in mean sGAG release compared with untreated controls (Figure 4a). The highest concentration of EPA, namely 10 μM, produced the greatest loss of sGAG, with statistically significant differences at all time points up to 96 hours. In the presence of IL-1β, rates of sGAG release were attenuated when explants were treated with 0.1 or 1 μM EPA (Figure 4b). The differences were statistically significant throughout the 120-hour treatment period. EPA at 10 μM, however, failed to attenuate the IL-1β effect at any time point except 120 hours. Even at 0.1 and 1 μM, EPA failed to reduce sGAG loss to the level found for control explants not treated with IL-1β such that the difference remained statistically significant.

Very similar trends were seen in identical experiments using DHA. A 10 μM DHA dose induced a small but statistically significant increase in sGAG release rate with respect to untreated controls at all time points (Figure 5a). The lower doses only had a statistically significant effect in the early stages of the treatment period. In the presence of IL-1β, DHA at 0.1 and 1 μM reduced sGAG loss. The differences between IL-1β-treated explants and those supplemented with DHA at 0.1 and 1 μM were statistically significant for up to 96 hours of treatment (Figure 5b). Indeed, in the first 24 hours, the effect was so great that there was no statistically significant difference (*P *> 0.05; Mann-Whitney *U *test) between untreated control explants and IL-1β-treated explants supplemented with 0.1 μM DHA. However, after 120 hours of treatment, DHA no longer had any effect on sGAG release in the presence of IL-1β such that there were no statistically significant differences between IL-1β-treated explants with and without DHA (*P *> 0.05; Mann-Whitney *U *test).

### True corrected efficacy of the n-3 fatty acids and efficacy with time

The efficacy of the fatty acid treatments was calculated at each time point using data that were first adjusted for the influence of the two vehicles, BSA and ethanol (Equation 1 below). Parameters indicate the mean fold increase over untreated control. Efficacy (%) =

$$\left\{\frac{\left[\left(IL-1\beta \right)-\left(BSA\right)\right]-\left[\left(fattyacidtreatment\right)-\left(BSA\right)-\left(ethanol\right)\right]}{\left[\left(IL-1\beta \right)-\left(BSA\right)\right]-1}\right\}\times 100$$

An efficacy of 100% indicates that the fatty acid reduces sGAG loss to that seen in untreated controls.

**Figure 6:**
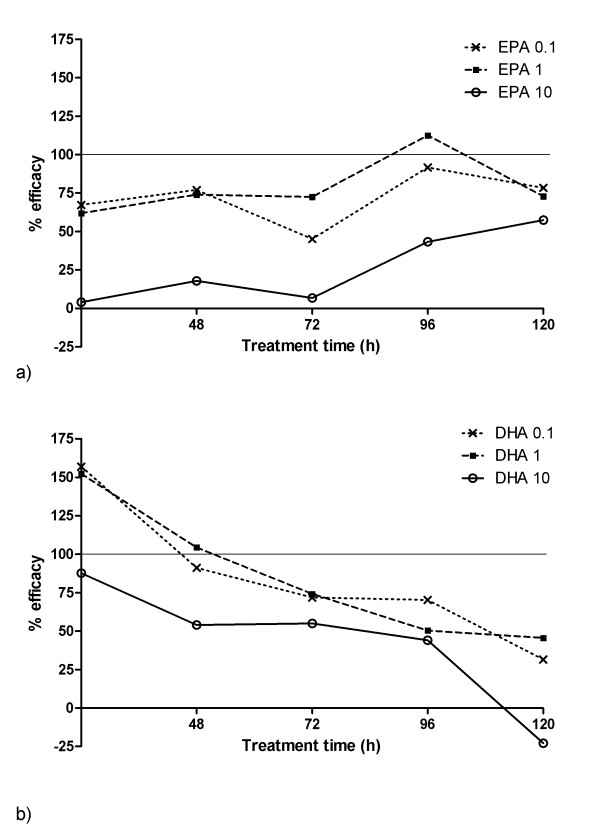
**EPA exhibits are more prolonged anti-catabolic efficacy compared to DHA**. Efficacy of EPA **(a) **and DHA **(b) **in terms of their ability to reduce sGAG loss from cartilage explants treated with 10 ng.mL^-1 ^IL-1β. See Results for calculation of percentage efficacy.

Figures 6a and 6b show the efficacy for EPA and DHA treatments, respectively, to view the efficiency trends with time. The data indicate that EPA at 0.1 and 1 μM maintains potent efficacy at approximately 75% throughout the 5-day treatment period. DHA efficacy was initially very high in the first 24 hours, reducing sGAG loss below that for untreated control, hence having an efficacy greater than 100%. However, efficacy was reduced substantially over 5 days for all three concentrations tested.

To examine the temporal changes in efficacy, a linear regression was fitted to the data for each treatment and a Pearson's correlation analysis was performed. The results, shown in Table 3, confirm that for all DHA treatments, there were statistically significant reductions in efficacy over the 5 days. By contrast, EPA treatment at 0.1 and 1 μM showed no statistically significant temporal changes in efficacy. At 10 μM, EPA showed a statistically significant positive correlation with an increase in efficacy from very low values in the first 72 hours of treatment to approximately 50% at 120 hours.

**Table 3:** Linear regression analysis of efficacy with time calculated using Pearson's correlation^a^

Concentration (μM)	EPA	DHA
0.1	ns	*P *< 0.05 ↓
1	ns	*P *< 0.01 ↓
10	*P *< 0.05 ↑	*P *< 0.05 ↓

^a^Data are also presented Figure 6. EPA, eicosapentaenoic acid; DHA, docosahexaenoic acid. ns, not significantly different. Direction of arrow refers to positive or negative correlation between efficacy and time.

## Discussion

This work investigated the potential therapeutic anticatabolic effects of EPA and DHA by directly measuring tissue degradation in a physiologically relevant, accelerated model of inflammatory joint disease. IL-1β treatment at 10 ng.ml^-1 ^as used in previous studies [13,26-28]) powerfully stimulated the release of sGAG over a 5-day period in accordance with an acute catabolic response [29]. By the end of the IL-1β treatment period, explants had released to the media approximately 15 μg.mg^-1 ^of sGAG, equivalent to 1-2% of total wet weight and an estimated 15-30% of total proteoglycan. When these data are normalised to untreated control values, it is clear that IL-1β-induced sGAG loss was greatest in the first 24 hours and decreased thereafter (Figure 3). Previous studies have reported that BSA induces cytokine secretion [30]. Indeed, in the present study, approximately 20-30% of the procatabolic effects of the IL-1β treatment can be attributed to the BSA, used here as a vehicle for the IL-1β.

Studies using chondrocytes in monolayer showed that a 24-hour predose of the n-3 compounds, EPA and DHA, reduced subsequent mRNA levels for degradative enzymes relative to IL-1β-stimulated levels [13]. However, using the explant model and measurement of sGAG release, preliminary studies found that pretreatment with EPA or DHA did not alleviate the subsequent catabolic effects of IL-1β (data not shown).

However, using the prolonged treatment protocol, EPA and DHA, at 0.1 and 1 μM, did provide a statistically significant attenuation of IL-1β-mediated tissue breakdown (Figures 4b and 5b). This occurred despite EPA and DHA inducing mild catabolic effects of their own in the absence of IL-1β, particularly at the higher dose of 10 μm (Figures 4a and 5a). These results support the hypothesis that oxidisation of these n-3 compounds gives rise to the mildly inflammatory PGE_3 _and LTB_5_, which, in the presence of IL-1β, competitively inhibit the arachidonic acid pathway [23]. It has additionally been postulated that EPA blocks the terminal stages of aracidonic acid synthesis from its precursors *in vivo *[31].

Mechanisms have also been proposed which implicate cell types beyond chondrocytes. One such example suggests that EPA-derived 15-lipoxygenase products interfere with nuclear factor (NF)-κB activation, preventing proinflammatory leukocyte adhesion receptor expression, and subsequent leukocyte-endothelial interactions [32]. In addition to this, studies, initially in the context of aspirin, have implicated a new potent anti-inflammatory EPA-derived mediator in the shape of the resolvin E1 and novel wider anti-inflammatory circuits [33,34]. Our *in vitro *model isolates the cartilage from systemic or other local surrounding influence so that the anticatabolic effects of EPA and DHA can only be attributable to competitive inhibition of arachidonic acid oxidation or other mechanisms mediated solely by chondrocytes. The therapeutic effects of these n-3 compounds may therefore be even more profound and longer lasting *in vivo*, where broader anti-inflammatory circuits can be engaged.

The compounds were highly effective in the early periods, during which the most acute IL-1β effects were seen. In the case of 0.1 and 1 μM EPA, efficacy was maintained at approximately 75% throughout the treatment period (Figure 6a). By contrast, DHA efficacy was initially greater than that for EPA but significantly reduced with time (Figure 6b). The reason for this declining efficacy is as yet unclear. To further elucidate any sustained mode of action for EPA and DHA, preliminary studies were conducted to examine the expression of catabolic inflammatory genes at the end of the 5-day treatment period. Using well-established quantitative polymerase chain reaction (qPCR) techniques previously described [35], gene expression for ADAMTS 4 and 5; MMPs 3, 9 and 13; and cyclooxygenase 2 (COX2) was assessed relative to untreated controls and normalised to 18S. IL-1β robustly raised all six genes consistent with its well-established mode of action (Table 4). Treatment with EPA reduced the IL-1β-mediated elevation of these catabolic genes with the exception of MMP9, which was unaffected. By contrast, for DHA at the 5-day time point, no such attenuations were observed. These data concur with the observed loss of efficacy displayed by DHA in the tissue degradation studies (Figure 6) and suggest that EPA at least produces its anticatabolic effect by downregulating catabolic gene expression.

**Table 4:** Analysis of gene expression at the end of the 5-day treatment period^a^

		ADAMTS4	ADAMTS5	MMP3	MMP9	MMP13	COX2
EPA	0.1 μM	↓	↓	↓	x	↓	↓
	1 μM	↓	x	↓	x	↓	↓

DHA	0.1 μM	x	x	x	x	x	x
	1 μM	x	x	x	x	x	x

^a^All six catabolic/inflammatory genes were upregulated in the IL-1β-treated explants relative to untreated controls. Arrows (↓) indicate a reduction in the IL-1β-induced increase in gene expression associated with EPA or DHA treatment. EPA, eicosapentaenoic acid; DHA, docosahexaenoic acid; MMPs, matrix metalloproteinases; COX, cyclooxygenase; ADAMTS, a disintegrin and metalloproteinase with a thrombospondin type 1 motif.

## Conclusions

In conclusion, previous studies using isolated chondrocytes have demonstrated that EPA and DHA reduce catabolic gene expression profiles [13], but this is the first study to robustly show in a cartilage explant model that these anticatabolic effects of PUFAs are translated into reductions in tissue degradation. The n-3 compounds, particularly EPA, which maintains its efficacy, have exciting therapeutic potential for the treatment of inflammatory joint diseases such as osteoarthritis.

## Abbreviations

AA: arachidonic acid; ADAMTS: a disintegrin and metalloproteinase with a thrombospondin type 1 motif; BSA: bovine serum albumin; COX: cyclooxygenase; DHA: docosahexaenoic acid; DMB: 1,9-dimethylmethylene blue; DMEM: Dulbecco's modified Eagle's medium; EPA: eicosapentaenoic acid; FCS: foetal calf serum; IL-1β: interleukin 1 beta; LTB: leukotriene B; MMPs: matrix metalloproteinases; OA: osteoarthritis; PGE: prostaglandin E; PUFAs: polyunsaturated fatty acids; sGAG: sulphated glycosaminoglycan; TIMPS: tissue inhibitors of metalloproteinases.

## Competing interests

The authors declare that they have no competing interests.

## Authors' contributions

AW participated in study design, acquisition of data, analysis and interpretation of data and manuscript preparation. JM participated in study design, acquisition of data and analysis and interpretation of data. EJB completed acquisition of gene expression data and was involved in subsequent interpretation of the data and manuscript preparation. AM-T participated in study design, interpretation of data, manuscript preparation and supplied n-3 compounds. MK participated in study design, analysis and interpretation of data, statistical analysis and manuscript preparation.
